# Inferring Crohn’s disease association from exome sequences by integrating biological knowledge

**DOI:** 10.1186/s12920-016-0189-2

**Published:** 2016-08-12

**Authors:** Chan-Seok Jeong, Dongsup Kim

**Affiliations:** Department of Bio and Brain Engineering, Korea Advanced Institute of Science and Technology (KAIST), 291 Daehak-ro, Yuseong-gu, 34141 Daejeon Republic of Korea

## Abstract

**Background:**

Exome sequencing has been emerged as a primary method to identify detailed sequence variants associated with complex diseases including Crohn’s disease in the protein-coding regions of human genome. However, constructing an interpretable model for exome sequencing data is challenging because of the huge diversity of genomic variation. In addition, it has been known that utilizing biologically relevant information in a rigorous manner is essential for effectively extracting disease-associated information.

**Results:**

In this paper, we incorporate three different types of biological knowledge such as predicted pathogenicity, disease gene annotation, and functional interaction network of human genes, and integrate them with exome sequence data in non-negative matrix tri-factorization framework. Based on the proposed method, we successfully identified Crohn’s disease patients from exome sequencing data and achieved the area under the receiver operating characteristics curve (AUC) of 0.816, while other clustering methods not using biological information achieved the AUC of 0.786. Moreover, the disease association score derived from our method showed higher correlation with Crohn’s disease genes than other unrelated genes.

**Conclusions:**

As a consequence, by integrating biological information across multiple levels such as variant, gene, and systems, our method could be useful for identifying disease susceptibility and its associated genes from exome sequencing data.

## Background

The advent of high-throughput sequencing technologies has enabled determining detailed catalogues of genomic sequence variants. Especially, cost-effective exome sequencing has been emerged for extending variant association studies to include rare variants [1]. In Crohn’s disease (CD), exome sequencing was adopted to identify the causative variants and the genes affected by them [2]. Despite that some studies have successfully identified CD associated variants and genes [3–5], the genetic heterogeneity and environmental effects on CD still obscure the interpretation of CD exome sequencing data. Particularly, since most of pathogenic variants are enriched for rare variants [6], a large amount of samples more than 10,000 exome sequences are required for the association study [7]. Furthermore, predicting disease susceptibility of exome sequence for clinical applications is still challenging.

To efficiently investigate the relationship between sequence variants and disease susceptibility, integrating variant-level and gene-level information is important [8]. Analogously, Na et al. [9] carried out ranking susceptible diseases for personal genome sequence by comparing gene-level pathogenicity vectors derived from genome sequence variants and disease-gene association knowledge, respectively.

In this study, we predict CD susceptibility from 56 exome sequences by integrating biological knowledge described at variant-level, gene-level, and systems-level. For the integrative analysis, we adopt the computational framework called non-negative matrix tri-factorization (NMTF) [10, 11], and introduce the constraints for deriving biologically relevant solution. This approach distinguishes the exomes of CD patients, and simultaneously prioritizes the corresponding CD associated genes. This unique feature could be beneficial for clinical applications based on personal genome interpretation.

## Methods

### Data set

We obtained exome sequencing data from the Crohn’s disease challenge of CAGI 2011 (https://genomeinterpretation.org). The purpose of the CAGI challenge was to distinguish exomes of Crohn’s patients and healthy individuals. The data is formatted in a variant call format (VCF), and the exome samples are randomly numbered. Besides the exome sequences, any other information is not given. The exomes were obtained from 56 individuals, consisting of 42 patients with Crohn’s disease and 14 healthy individuals. From the exome sequences, a total of 155,019 coding DNA sequence variants, resulting in 1202 nonsense, 79,448 nonsynonymous, and 74,577 synonymous mutations, are identified. For the present work, we used the nonsynonymous mutations of 33,948 amino acid substitutions of 11,435 human genes.

To distinguish Crohn’s disease patients from the exomes, we utilized various biological information. First, pathogenicity of amino acid substitutions predicted using PolyPhen-2 [12]. Second, knowledge on disease-related genes was collected from DGA database [13]. We obtained 189 genes associated with Crohn’s disease (DOID: 8778) on March 2013. Third, knowledge on functional interactions between human genes was collected from HumanNet [14]. We downloaded a functional gene network from the HumanNet website, and selected only the genes corresponding to the genes of the above exome data set. Consequently, 151,440 gene-gene interactions of 9597 human genes were obtained.

### Non-negative matrix tri-factorization

**Fig. 1:**
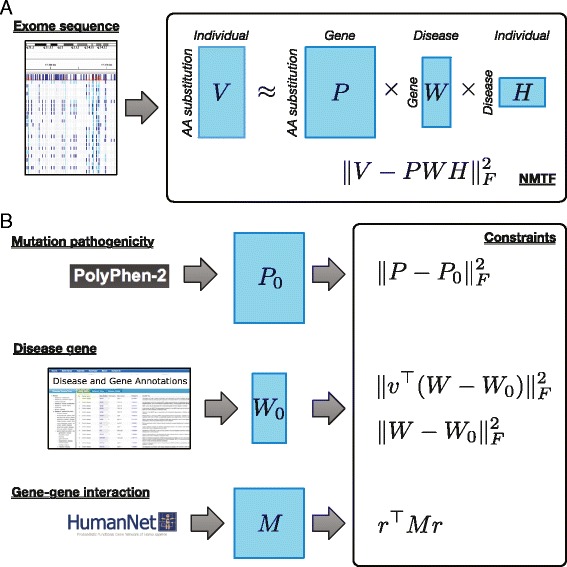
Overview of NMTF model and constraints for distinguishing disease exome and identifying disease-associated genes. **a** Exome sequences are represented as the matrix *V* encoding the occurrences of amino acid substitutions. *V* is approximated by the multiplication of three different matrices *P*, *W*, and *H*, which represent pathogenicity of mutations, disease-associated genes, and disease status of exomes, respectively. **b** To obtain a biologically meaningful solution, three kinds of biological knowledge, such as pathogenicity prediction, known annotation of disease genes, and functional interaction between genes, are integrated as constraints

Because of a huge of diversity of genomic variations, inferring disease-exome association is very challenging. For that reason, an integrated method that utilizes different kinds of biological knowledge and bioinformatics predictions would be effective. We adopted NMTF to integrate various information as illustrated in Fig. 1a. The notations and definitions used here are listed in Table 1. NMTF tri-factorizes an input non-negative matrix into three different non-negative matrices, whose multiplication approximates the input matrix. This can be written as

$$V \approx PWH \text{.} $$

*V* is a *n*×*m* binary matrix with 1 for amino acid substitution occurrence and 0 otherwise. *P*, *W*, and *H* are *n*×*l*, *l*×2, and 2×*m* factorized matrices, respectively. *P* represents pathogenic effects of amino acid substitutions, *W* represents CD associations of genes, and *H* represents CD association of individual exomes. For convenience, we assume that the first column of *W* and the first row of *H* correspond to CD, while the second column and the second row indicate healthy status, respectively.

**Table 1:** Notations

Notation	Definition
*n*	Number of amino acid substitutions
*m*	Number of exomes
*l*	Number of genes
*l*_*D*_	Number of known Crohn’s disease genes
*V*	Exome matrix (*n*×*m*)
*P*	Pathogenicity of amino acid substitutions (*n*×*l*)
*W*	Disease-gene association matrix (*l*×2)
*H*	Disease-exome association matrix (2×*m*)
*M*	Gene-gene interaction network (*l*×*l*)
*P*_0_	Pathogenicity prediction by PolyPhen-2 (*n*×*l*)
*W*_0_	Annotated Crohn’s disease-gene association matrix (*l*×2)
*v*	Indicator vector for known Crohn’s disease genes (*l*)
*r*	Crohn’s disease association vector derived from *W* (*l*)

To represent the CD associations of each gene and exome, *W* and *H* are normalized as

$$\sum_{j=1}^{2} W_{ij} = 1 \text{and } \sum_{i=1}^{2} H_{ij} = 1 \text{.} $$

Due to the non-negativity constraint, *W*_*i*1_ is ranged from 0 to 1, where 1 means that the gene *i* is associated with CD. In the same manner, *H*_1*j*_ is ranged from 0 to 1, where 1 means that the exome *j* is associated with CD.

*P*, *W* and *H* can be derived by minimizing the squared error between the original and the reconstructed matrices, which can be written as

$$\min_{P, W,\, H \geq 0} \| V-PWH {\|_{F}^{2}} \text{.} $$

However, the optimization equation often does not have a unique solution, and could be sensitive to the noise in the data and the algorithm used for finding the optimal solution.

### Constraints for integrating biological knowledges

To derive the tri-factorization solution biologically meaningful, we introduce three sorts of constraints based on heterogeneous biological information as shown in Fig. 1b.

First, to preferentially address disease-causing mutations, the mutation pathogenicity constraint is introduced as

$$\min \| P-P_{0} {\|_{F}^{2}} \text{,}$$

where *P*_0_ represents the predicted pathogenicity of amino acid substitution. The predicted pathogenicity is obtained by running PolyPhen-2. We determine (*P*_0_)_*ij*_ as the PolyPhen-2 prediction value if the amino acid substitution *i* belongs to the gene *j*. Otherwise, 0 is assigned. This constraint enforces to prioritize disease-causing mutations more than neural variants.

Second, we utilize the annotation of known disease-associated genes, and introduce the disease gene constraint as

$$\min \| W-W_{0} {\|_{F}^{2}} \text{,}$$

where *W*_0_ represents known CD-associated genes collected from DGA database. (*W*_0_)_*i*·_ is determined as [1 0] if the gene *i* is annotated as CD-associated gene. Otherwise, [0.5 0.5] is assigned. To consider that a relatively small number of CD genes are annotated among *l* genes, we enforce CD genes by using the following constraint defined as

$$\min \| v^{\top} (W-W_{0}) {\|_{F}^{2}} \text{,}$$

where *v* is an indicator vector for CD genes, *i*.*e*., *v*_*i*_ is determined as 1 for CD gene and 0 otherwise.

Third, we introduce the gene-gene interaction constraint, which enforces functionally interacting genes to be simultaneously clustered. Because functionally interacting genes perform for the similar phenotypes, disease genes could interact with each other through the functional interaction network. To address the functional relationship between CD genes, we use the constraint defined as

$$\max r^{\top} Mr \text{,}$$

where *r* is CD association score vector derived from *W* as

$$r = [W_{11} \ W_{21} \ W_{31} \ \cdots \ W_{l1}]^{\top} \text{,}$$

and *M* encodes functional interactions obtained from HumanNet. The HumanNet describes functional interactions as a probabilistic value from 0.6 to 1.0. *M*_*ij*_ is determined as the interaction probability between the genes, *i* and *j*. For the gene pairs discarded in HumanNet due to low interaction probability, 0 is assigned. Therefore, this constraint term has a higher value as interacting genes are clustered together.

### Optimization procedure

The NMTF squared error term and the constraint terms are combined and formulated as the objective function defined by

$$\begin{aligned} {}\min_{P, W,\, H \geq 0}& \| V-PWH {\|_{F}^{2}} + \alpha \| P-P_{0} {\|_{F}^{2}}\\&+ \beta \| v^{\top} (W-W_{0}) {\|_{F}^{2}} - \gamma r^{\top} Mr + \lambda \| W-W_{0} {\|_{F}^{2}} \text{,} \end{aligned} $$

where *α*, *β*, *γ*, and *λ* represent the weight parameters for the constraint terms.



To find the optimal solution for the objective function, we used the multiplicative update algorithm [15], because it is simple to implement and usually performs well. Our optimization algorithm is described in Algorithm 1. The algorithm initializes the factorized matrices *P*, *W*, and *H* with random non-negative values. Then, each matrix is iteratively updated with fixing the other matrices, until the algorithm converges. Since the multiplicative update algorithm achieves a local optimum, we repeated the computation 100 times with different initial matrices, and selected 30 solutions with smallest squared errors. Then, the final solution was obtained by averaging them over the replicas.

## Results

### Selecting NMTF models

**Fig. 2:**
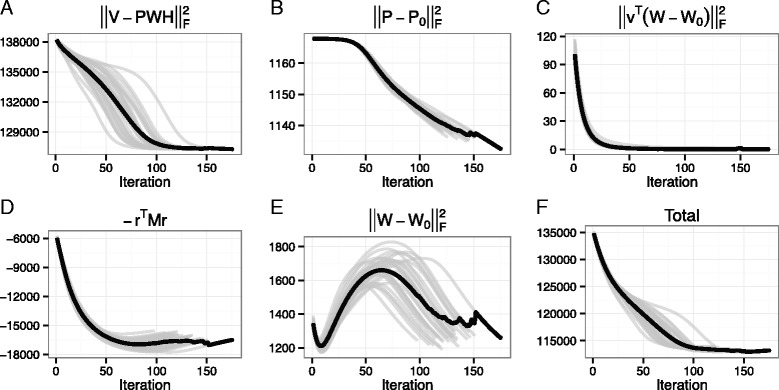
Convergence curve of NMTF optimization procedures. **a** The squared error, **b** the mutation pathogenicity constraint, **c** and **e** the disease gene constraint, **d** the gene-gene interaction constraint, and **f** the total values of objective function are denoted on the y-axis. The x-axis denotes the iteration number. The *black line* represents the averaged line over the best-30 optimization results encoded as *grey lines*

In our objective function, the hyper-parameters weighting constraint terms should be properly chosen. We performed the optimization with different hyper-parameters, and compared the squared errors of the resulting solutions. The hyper-parameter *α*, *β*, *γ*, and *λ* were searched in {0.05, 0.1, 0.2}, {1, 2, 4}, {0.05, 0.1, 0.2}, and {0.5, 1, 10, 15}, respectively. When comparing the squared errors, we found similarly good approximations and convergence timings with *α*, *β*, *γ*, and *λ* in {0.05, 0.1}, {1, 2}, {0.1, 0.2}, and {0.5, 1}, respectively. In the following results, we used 0.1, 1, 0.1, and 1 for *α*, *β*, *γ*, and *λ*, respectively. By using the chosen hyper-parameters, we repeated the NMTF optimization procedure 100 times with different initial solution matrices. Lastly, the final solution was obtained by averaging 30 solution matrices of the lowest squared errors. The squared error and constraint values and the total values of objective function are shown in Fig. 2. The replicas were consistently converged in 98–176 iterations (129.7 iterations on average), and the resulting scores of objective function showed high correlation.

### Distinguishing Crohn’s disease patients and healthy individuals

**Fig. 3:**
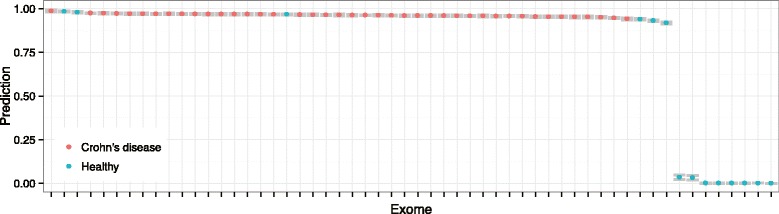
Prediction of exomes for Crohn’s disease patients. The x-axis denotes each exome, and the y-axis denotes the prediction value averaged over the best-30 NMTF solutions. The error bars are encoded grey. The Crohn’s disease patients and the healthy individuals are encoded with different colors

We identified the exome sequences of CD patients by using the solution matrix *H*. Since we bound *H* in [0, 1], the prediction values is also bounded in [0, 1]. One indicates that the exome belongs to CD patient, and 0 indicates that it belongs to healthy individual. Fig. 3 shows the prediction results for 56 exome sequences. Most of the predictions are close to 1 or 0. In addition, they show a small variation over replicas, indicating that the solution matrice *H* of replicas are highly correlated. For CD patients, all their exomes are classified as CD, but, for healthy individuals, 8 among 14 exomes are correctly classified as healthy. Although 6 healthy individual exomes are misclassified to CD, three of them show smaller prediction values than CD patient exomes. We find that the distributions of prediction values for CD patients and healthy individuals are significantly different from each other (two-tailed Mann-Whitney U-test, *p*-value = 2.45×10^−4^).

**Fig. 4:**
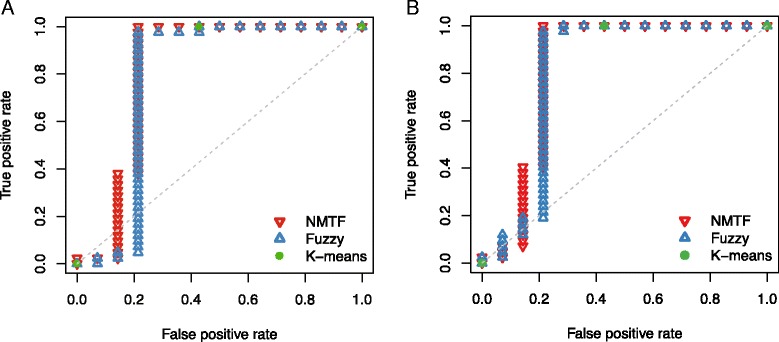
ROC plots of NMTF (*red*), fuzzy clustering (*blue*), and *k*-means clustering (*green*) methods incorporating (**a**) PolyPhen-2 and (**b**) SIFT

Because our prediction for an exome matches to its soft membership in a specific cluster, we compared the predictive performance with other clustering methods, such as *k*-means and fuzzy clusterings. We represented each exome as a vector identical to the column vector of *V* matrix, and clustered the exomes to two clusters. Although they did not give an interpretable annotation for each cluster, we assumed that the bigger cluster represented CD because the number of CD exomes exceeded that of healthy in out data set. In addition, the membership probability is used as prediction value for fuzzy clustering. We compared the receiver operating characteristic (ROC) curves for predicting CD patient exomes as shown in Fig. 4a. NMTF showed better area under the ROC curve (AUC) of 0.816, while *k*-means and fuzzy clusterings showed the AUCs of 0.786. We found that the 8 healthy individuals easily discriminated by NMTF were clustered in a group by both clustering methods. Since the 8 healthy individuals were easily classified, we excluded them and estimated AUCs for the remaining 42 CD and 6 healthy exomes. As *k*-means did not provide membership probability, we only compared NMTF and fuzzy clustering for the other healthy individuals and CD patients. When comparing the AUCs, NMTF performed better with AUC of 0.571 than fuzzy clustering with AUC of 0.5. In addition, we evaluated the performance of NMTF by averaging the best-10, 20, 40, and 50 solution matrices, but the AUCs were ranged in 0.532–0.564, still outperforming fuzzy clustering. Consequently, the results indicate that the biological knowledges integrated in NMTF framework were useful for distinguishing exome sequences of CD patients from healthy individuals.

In exome sequencing studies, SIFT [16] is one of the most highly used tools, as well as PolyPhen-2, to predict the functional consequences of nonsynonymous variants. Thus, we performed the NMTF analysis by replacing the predicted pathogenicity of PolyPhen-2 with that of SIFT. Because the prediction of SIFT web-server was available for 15,810 amino acid substitutions among our data set, we only used those variants. As shown in Fig. 4b, NMTF showed better AUC of 0.806, while *k*-means and fuzzy clusterings show AUCs of 0.786 and 0.793, respectively. Also, the prediction values of NMTF for CD patients are significantly higher than those for healthy individuals (*p*-value = 4.09×10^−4^). Although the use of PolyPhen-2 achieved higher AUC value than the use of SIFT, it may be caused by better performance of PolyPhen-2 [17]. As a consequence, the predicted pathogenicity utilized in NMTF framework could be derived from various predictors such as MutationTaster [18], FATHMM [19], PANTHER [20], GERP++ [21], PhyloP [22], and so on.

**Fig. 5:**
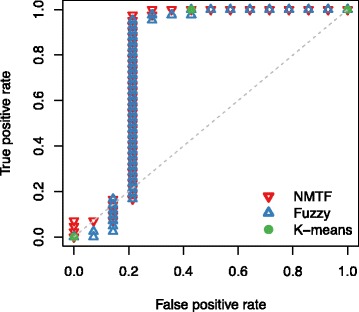
ROC plots of NMTF (*red*), fuzzy clustering (*blue*), and *k*-means clustering (*green*) methods for rare variant data set

Although many studies using exome sequencing have aimed to identify rare coding varaiants causative in complex diseases, analyzing the rare variants is still challenging because of the small sample size. To address this issue, we excluded commonly occuring variants, and performed the NMTF analysis for the remaining variants. Common variants, with the minor allele frequencies of > 0.01 and not annotated as diasese causing, were extracted from Ensembl database [23]. Then, 18,999 variants were used for predicting CD patients. As shown in Fig. 5, NMTF showed AUC of 0.821, outperforming *k*-means and fuzzy clusterings with AUCs of 0.786 and 0.808, respectively. Also, the prediction values of NMTF significantly differs between CD patients and healthy individuals (*p*-value =1.88×10^−4^). Therefore, the NMTF framework can be used for analyzing exome sequences based on rare variants.

### Analysis of Crohn’s disease-associated genes

**Fig. 6:**
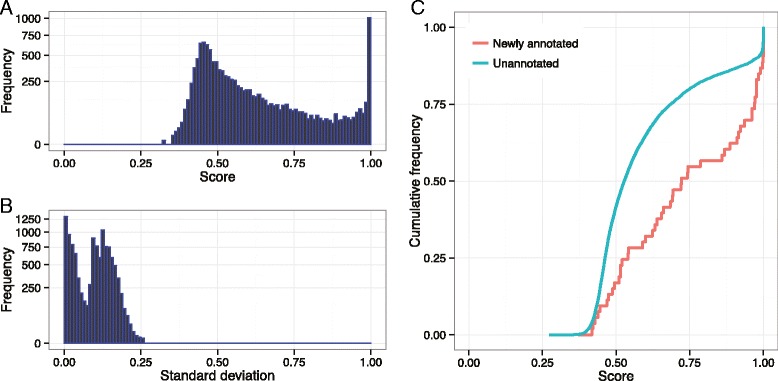
Distribution of Crohn’s disease association scores. **a** Distribution of CDA scores of genes derived from the solution matrix *W*. The scores are calculated by averaging the best-30 NMTF solutions. **b** Distribution of standard deviations of CDA scores of each gene. By the definition of standard deviation, the value cannot exceed 0.509, because the CDA score is ranged from 0 to 1. **c** Cumulative distribution of CDA scores for Crohn’s disease genes and other unknown genes. Note that the newly annotated and unannotated genes are identically constrained in the disease gene constraint metrix *W*_0_

We examined the CD association of genes by analyzing the solution matrix *W*. Similar to *H*, the CD association (CDA) scores of *W* are ranged in [0, 1], such that the score of 1 represents strong CD association, and the score of 0.5 represents neutral association, as encoded in the constraint matrix *W*_0_. The CDA scores were distributed as shown in Fig. 6a. The known CD genes encoded in *W*_0_ had the scores close to 1 (0.998 on average). On the other hand, the other genes had the scores widely ranged in 0.326–1.0, showing the average score of 0.602. We investigated the variation of CDA scores over replicas, but most of genes showed small variations as shown in Fig. 6b.

To investigate the correlation of CDA score and CD gene, we collected CD genes from the DGA database on July 2015, and selected newly annotated CD genes, not used in *W*_0_. We obtained 53 newly annotated CD genes, and examined their CDA scores in comparison with those of the other unannotated genes, as shown in Fig. 6c. The distribution of CD genes were shifted close to 1. For the newly annotated CD genes, 15.1 % and 30.2 % of genes showed the CDA scores greater than 0.99 and 0.95, respectively. Whereas, for the other genes, only the 8.9 % and 11.6 % of genes showed the CDA scores in the same ranges, respectively. Therefore, CDA score derived by NMTF could be informative for inferring disease-gene relationship.

## Discussion and conclusion

In this study, we developed a computational framework called NMTF for analyzing exome sequencing data, and integrated biological knowledge relevant to the disease susceptibility. By applying the proposed method to 56 exome sequences, we discriminated the exomes of CD patients and healthy individuals, and demonstrated the correlation between CD genes and CDA scores.

This study makes two major contributions to the exome sequencing data analysis. First, our method, in which disease-associated individuals and genes are interconnected by co-clustering, provides an interpretable analysis for cliniical decision making. For example, an additional information connecting the disease susceptibility to the evident genes can be derived. Although the compared clustering methods showed a certain degree of predictive performance for the CD data set, they lack the interpretability. On the other hand, in our method, co-clustered genes in our method could support the genetic basis determining the CD susceptibility of exomes. This would be beneficial for understanding the heterogeneity of genetic effects in genetically complex disease, and designing effective personalized treatments.

Second, we demonstrated that integrating multi-level information could be useful for understanding genetically complex diseases. Based on the NMTF framework, we combined a wide range of biological information including the predicted pathogenicity of single amino acid substitution, the annotation of disease-gene association, and the functional interaction between human genes. By doing so, we inferred the disease information from the variant-level data. Although Na et al.’s study [9] showed the integration of variant-level and gene-level information, their approach requires well-curated knowledge on disease-gene association. However, our approach is designed to complement imperfect prior-knowledge on disease-gene association, by using the systems-level information, functional interaction of human genes, as disease-associated genes often share common biological functions [24]. The integration of multi-level information may be effective because CD susceptibility is affected by complicated genetic regulations and interactions. Similarly, this approach would be useful for other complex diseases in the same manner with CD.
